# Pan-RAS Inhibitors: Expanding Therapeutic Potential and Evading Resistance

**DOI:** 10.3390/cancers18111844

**Published:** 2026-06-04

**Authors:** Sindhu Ramesh, Junwei Wang, Chung-Hui Huang, Austin M. Moore, Khalda Fadlalla, Kristy L. Berry, Yulia Y. Maxuitenko, Xi Chen, Adam B. Keeton, Bassel El-Rayes, Donald J. Buchsbaum, Karim I. Budhwani, Gang Zhou, Amit K. Mitra, Gary A. Piazza

**Affiliations:** 1 Department of Drug Discovery and Development, Harrison College of Pharmacy, Auburn University, Auburn, AL 36849, USA; szr0065@auburn.edu (S.R.); jzw0164@auburn.edu (J.W.); czh0113@auburn.edu (C.-H.H.); amm0117@auburn.edu (A.M.M.); kaf0009@auburn.edu (K.F.); klb0131@auburn.edu (K.L.B.); yym0001@auburn.edu (Y.Y.M.); xzc0059@auburn.edu (X.C.); abk0039@auburn.edu (A.B.K.); akm0060@auburn.edu (A.K.M.); 2 Division of Oncology, Moores Cancer Center, University of California, San Diego, CA 92037, USA; belrayes@health.ucsd.edu; 3 CerFlux, Inc., Birmingham, AL 35203, USA; djb@uab.edu (D.J.B.); ironman@cerflux.com (K.I.B.); 4 Department of Obstetrics and Gynecology, University of Alabama at Birmingham, Birmingham, AL 35233, USA; 5 Georgia Cancer Center, Medical College of Georgia, Augusta University, Augusta, GA 30912, USA; gzhou@augusta.edu

**Keywords:** cancer, pan-RAS, pan-KRAS, RAS, resistance, therapeutic

## Abstract

Approximately 30% of all human cancers are driven by mutations in *RAS* genes, *KRAS, HRAS*, and *NRAS*, resulting in the constitutive activation of RAS proteins and stimulation of MAPK/AKT signaling. Non-mutant, i.e., wild-type (WT) RAS can also become activated through mechanisms such as gene amplification or excessive stimulation by mutated or overexpressed receptor tyrosine kinases (e.g., EGFR), thereby promoting cancer progression. Mutant or activated RAS contributes to multiple hallmarks of cancer, including unchecked cellular proliferation, reprogrammed cellular metabolism, immunosuppression, and metastasis. Hence, RAS is of immense clinical importance, with hundreds of laboratories studying various aspects of RAS biology or developing RAS inhibitors. There is perhaps no greater unmet medical need in oncology than the need for a broadly efficacious but safe inhibitor of mutant and activated RAS. Mutant-specific KRAS G12C inhibitors have shown promising therapeutic efficacy, leading to FDA approval of sotorasib and adagrasib, although their use is limited to patients with the relatively rare G12C KRAS mutation. Mutant-specific KRAS inhibitors are also susceptible to adaptive resistance, in part, due to secondary *RAS* mutations, and compensatory signaling from WT RAS isozymes. A pan-RAS inhibitor capable of blocking all RAS isozymes, regardless of the underlying mutation, offers the potential for broader efficacy and capacity to avert resistance. While just a few years ago, pan-RAS inhibitors were predicted to be severely toxic or even fatal, the apparent safety profile of RMC-6236 (daraxonrasib), a pan-RAS inhibitor currently in clinical trials, suggests otherwise. Indeed, pan-RAS inhibitors are now considered by many in the RAS field to be the most promising class in development. In this review, we summarize the evolution and current status of pan-RAS and pan-KRAS inhibitors in preclinical and clinical development and highlight emerging human-relevant tumor models that are advancing preclinical evaluation.

## Simple Summary

About one-third of human cancers are caused by changes in a group of genes called RAS. These changes keep RAS proteins permanently “on,” driving cell growth and survival. Even normal (non-mutated) RAS can be overactivated by other cancer-related signals, further helping tumors to grow and spread. Because RAS plays such a central role, scientists have long tried to block it, but this has been difficult. Some newer drugs work for a specific type of RAS mutation, but only a small number of patients have this mutation, and tumors can quickly become resistant. A new type of drug called “pan-RAS inhibitors” is designed to block all forms of RAS, regardless of mutation. Early results suggest these drugs may be more effective and safer, offering hope for broader cancer treatment and reduced resistance. In this review, we summarize progress in these treatments and highlight improved versions of the models used to study them.

## 1. Introduction

**Figure 1:**
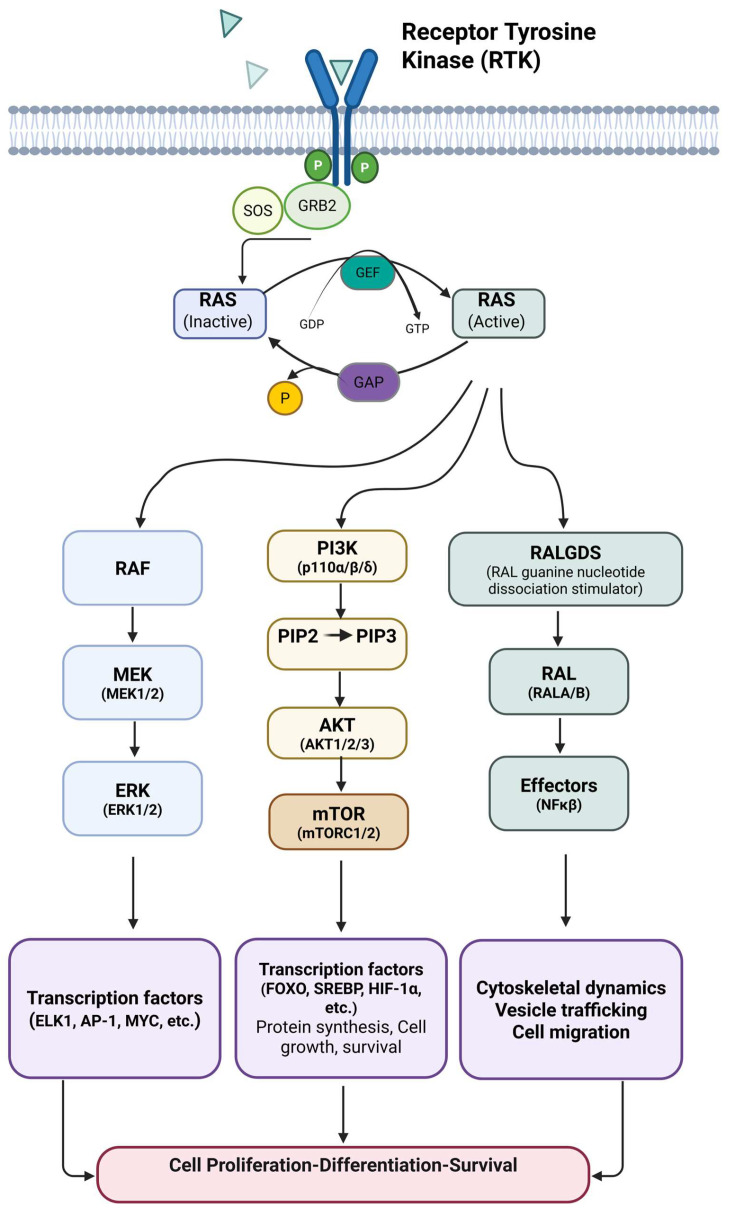
The RAS signaling pathway relays growth signals from activated growth factor receptors to the nucleus. The RAS/RAF/MEK/ERK pathway and the PI3K pathway are the classical RAS signaling pathways implicated in growth factor-mediated cell proliferation, differentiation, and cell death. Activation of the signaling network commonly occurs in human cancers through mutation of the components (TKR, RAS, RAF, PI3K and/or AKT or their negative regulators).

The *RAS* (rat sarcoma virus) family of oncogenes was first described in 1967 when mouse erythroblastosis virus (MEV) was used to induce lymphoma in rats [1]. Later, in the 1980s, *RAS* became the first cellular oncogene to be isolated [2] and it is the most frequently mutated oncogene in human cancer overall [3,4]. RAS is a membrane-bound small GTPase that, when bound to GTP, activates multiple effectors (RAF, PI3K, and others) to stimulate complex downstream signaling pathways, most notably the MAPK and AKT pathways that induce the transcription of proteins essential for cancer cell proliferation and survival (Figure 1) [5]. Under physiological conditions, RAS exists in cells in three distinct conformational states: (1) GTP-bound (“ON”), (2) GDP-bound (“OFF”), and (3) nucleotide-free (“Apo”) [6]. In the inactive form (“off” state), RAS is GDP-bound. GDP is dissociated by interaction with a Guanine Nucleotide Exchange Factor (GEF, e.g., SOS1) resulting in the apo state of RAS, which in turn transitions to the active form (“on” state) when GTP is bound. GTP-bound RAS can also be phosphorylated, rendering it inactive in the GTPase cycle [7,8]. Mutated RAS proteins are resistant to GTP hydrolysis, which renders them predominantly active (GTP-bound state), and persistently transmit deregulated signals to the cell nucleus, causing uncontrolled cellular proliferation in cancers. RAS mutants, particularly “fast exchange” mutants, may transition through the Apo state more frequently, thereby providing a potential opportunity to pharmacologically target this state of RAS [6]. While the RAS superfamily of GTPases comprises numerous members, Kirsten rat sarcoma virus (*KRAS*), neuroblastoma rat sarcoma virus (*NRAS*), and Harvey rat sarcoma virus (*HRAS)* are considered the canonical RAS oncogenic isoforms [9]. Among these, *KRAS* is the most frequently mutated in human cancers, accounting for approximately 85% of pancreatic ductal adenocarcinoma (PDAC), 37% of colorectal cancer (CRC), 30% of non-small cell lung cancer (NSCLC), and 26% of relapsed/refractory multiple myeloma (MM) cases [10,11,12].

Somatic gain-of-function mutations in *RAS* occur most frequently at amino acid residues G12, G13, and Q61. Mutations at these “hotspots” account for 99% of all *RAS* mutations. Among *KRAS* mutations, G12 mutations predominate (81%), followed by G13 (14%) and Q61 (2%). Among *HRAS* mutations, Q61 mutations are the most prevalent (38%), followed by G12 mutations (26%) and G13 mutations (23%). Among *NRAS* mutations, Q61 mutations are the most common (62%), followed by G12 (23%) and G13 (11%) [13]. Substitution of glycine by any amino acid other than proline at G12 or G13 causes a steric clash that prevents the GTPase-activating protein (GAP) arginine finger from catalyzing RAS-GTPase activity, thereby preventing the hydrolysis of GTP to GDP, and resulting in constitutive activation of RAS [14]. While these are the most common mutational frequencies overall, specific mutations can vary significantly across different cancer types. Mutations in *NRAS* and *HRAS* also play critical roles in oncogenesis. For example, NRAS mutations are frequently observed in hematological malignancies [15] and melanomas [16]. Among melanomas, approximately 80% of NRAS mutations occur at codon 61 [17]. Although Q61 mutations in KRAS are infrequent, these mutations are associated with the worst outcomes in PDAC [18]. While *HRAS* only accounts for around 1% of *RAS* mutations, there is a high prevalence of mutations at codon 12 in head and neck squamous cell carcinomas [19]. Further, Q61 is the most common *HRAS* mutation in urothelial carcinoma and the Follicular variant of papillary thyroid cancer [20,21].

RAS was long considered “undruggable” because of its high affinity for GDP and GTP, which made it technically challenging to develop nucleotide-competitive inhibitors. Furthermore, RAS lacks deep surface hydrophobic pockets, rendering the identification of high-affinity allosteric inhibitors challenging [11]. To overcome these obstacles, Shokat and colleagues introduced an alternative approach in 2013 that targeted the reactive cysteine-12 of KRAS G12C in its inactive (OFF) state using a covalent inhibitor [22]. The thermodynamic equilibrium between ON and OFF states was shifted by covalent engagement of the mutated cysteine, enabling irreversible stabilization of the inactive OFF-state and sustained downstream signaling suppression [23]. A covalent modification of Cys12 was predicted to disrupt KRAS G12C-driven signaling by permanent deactivation of the protein. This approach could overcome the inability of reversible inhibitors to block KRAS activation while enabling selective growth inhibition of KRAS-mutant cancer cells and sparing normal cells, potentially mitigating the toxicity concerns predicted to arise from non-selective inhibition of KRAS-driven cell growth.

As a result of Shokat’s discovery, sotorasib (AMG-510, brand name Lumakras), the first KRAS-targeted drug, was approved by the FDA in 2021 for the treatment of KRAS G12C mutant NSCLC and later for metastatic CRC. Sotorasib forms a covalent bond with the cysteine encoded by the KRAS G12C missense mutation when bound to GDP, locking the protein in the “off” state [24]. In clinical trials leading to FDA approval, patients treated with sotorasib had significantly longer progression-free survival (PFS) (5.6 months) compared to patients treated with docetaxel (4.5 months) after progression on a PD-1 or PD-L1 inhibitor (Hazard Ratio (HR) 0.66, 95% Confidence Interval (CI): 0.51–0.86). The longer PFS with sotorasib was also associated with fewer serious (grade ≥3) treatment-related adverse events compared with docetaxel (11% vs. 23%) [25].

Adagrasib (MRTX-849, brand name Krazati) was approved by the FDA in 2022 and, like sotorasib, is a covalent inhibitor of the KRAS G12C cysteine when GDP is bound to KRAS in the “off” state. Adagrasib treatment was associated with a median PFS of 6.5 months, a duration of response of 8.5 months, and an objective response rate (ORR) of 43%, as well as overall survival of 12.6 months, leading to FDA approval for adult patients with locally advanced or metastatic NSCLC [26]. While these results were comparable to sotorasib, adagrasib demonstrated superior efficacy in patients with brain metastases (ORR 33% vs. 25%) [26,27].

Despite the promising activity observed in clinical trials, a high percentage of patients treated with sotorasib or adagrasib were reported to develop resistance [28]. While secondary *RAS* mutations have been established as a common cause of resistance, non-genomic mechanisms, such as compensation from WT RAS isozymes, reactivation of MAPK/AKT signaling, and epithelial-to-mesenchymal transition, likely also contribute to resistance [29]. Hence, intensive research has been directed at identifying the mechanisms of acquired resistance, with the goal of developing novel inhibitors that provide sustained efficacy and confer a more durable survival benefit, especially in the context of drug-resistant metastatic malignancies. One strategy that emerged is to develop isozyme-specific KRAS inhibitors that act regardless of the mutational status. Pan-KRAS inhibitors have the potential to escape resistance, as patients who acquire resistance to mutant-specific G12C KRAS inhibitors may acquire additional *KRAS* mutations [28].

Another approach is the development of pan-RAS inhibitors that inhibit all three oncogenic RAS isozymes, KRAS, NRAS, and HRAS, regardless of mutational status. Potential advantages of pan-RAS inhibitors include their ability to treat a broader range of RAS-driven cancers, address the heterogeneity of RAS mutations, and prevent or delay the development of resistance arising from co-occurring RAS mutations or compensatory signaling by WT RAS isozymes. The scope of this review is to highlight the pan-RAS and pan-KRAS inhibitors currently in preclinical and clinical development, and to discuss various approaches that could result in broader efficacy and greater potential to evade resistance, which are major limitations of FDA-approved KRAS G12C inhibitors and, predictably, of other mutation-specific KRAS inhibitors in development.

## 2. Pan-RAS Inhibitors

Pan-RAS inhibitors act broadly across all isoforms (KRAS, HRAS, and NRAS) by targeting wild-type and/or mutant isoforms. Thus, pan-RAS inhibitors are predicted to achieve sustained tumor growth inhibition and broader activity across RAS-driven tumors by providing durable pathway suppression. These agents may offer an advantage in preventing adaptive resistance mediated by wild-type RAS isoforms. However, this mechanism also raises the risk of on-target toxicity in normal tissues due to inhibition of wild-type RAS signaling, as observed with RAS (ON) inhibitors developed by Revolution Medicines. These findings suggest that on-target toxicity may ultimately limit the achievable exposure and degree of RAS inhibition in patients treated with pan-RAS inhibitors versus isoform-specific inhibitors [30]. In this section, we provide an overview of emerging pan-RAS inhibitor therapeutic approaches as opposed to mutant-specific inhibitors, focusing on preclinical and preliminary clinical data supporting these innovative approaches.

### 2.1. Daraxonrasib (RMC-6236)

Daraxonrasib (RMC-6236) is the first pan-RAS inhibitor to enter clinical trials [31]. RMC-6236 belongs to a family of RAS inhibitors that share a common mechanism of action and are generally referred to as tri-complex inhibitors. RMC-6236 is specifically referred to as a tri-complex RAS^MULTI^ (ON) inhibitor. Since RAS is predominantly active in the “ON” state in cancer, binding directly to this configuration allows for more effective inhibition of the oncogenic driver. The compound family was originally derived from the natural product, sanglifehrin A, which binds cyclophilin A (CYPA) with high affinity. Modeling the CYPA structure after RMC-6236 binding to form a binary complex revealed a strong affinity for inhibiting activated RAS. The majority of RAS oncoproteins with missense mutations can be inhibited non-covalently by forming a tri-complex with CYPA. The resultant CYPA–inhibitor–RAS tri-complex interferes with downstream signaling by sterically inhibiting RAS–effector interactions.

RMC-6236 is currently in clinical trials with a mechanism of action similar to RMC-7977, which was initially developed as a tool compound to inhibit all three RAS isozymes, including those with a mutation at codon 12 (RAS G12X) and WT RAS. RMC-7977 demonstrated increased potency against KRAS G12X mutant cell lines compared to non-G12 mutant KRAS cell lines. Various oncogenic KRAS mutations exhibit distinct biochemical properties that contribute to increased drug sensitivity of KRAS G12X mutant cell lines [32,33]. Codon 13 and codon 146 mutations enhance nucleotide exchange, activating RAS through faster GDP-GTP cycling [34]. Primarily occurring in patients with CRC, codon 13 mutations are often co-mutated with *NF1* or *RTK* genes, thereby influencing RAS dependence and sensitivity to RMC-7977. Pharmacokinetic (PK) and pharmacodynamic (PD) studies of RMC-7977 in mice demonstrated a relationship between tumor concentrations of the drug and inhibition of the RAS pathway transcriptional target, DUSP6 [31]. Notably, the tumor concentrations of RMC-7977 were higher than the blood concentration in mouse xenograft models [31]. At an oral dose of 10 mg/kg, RMC-7977 was well tolerated and resulted in tumor growth inhibition and tumor regression across various panels of PDAC, CRC, and NSCLC cell-derived xenograft (CDX) and patient-derived xenograft (PDX) models bearing KRAS G12X mutations with a negligible impact on body weight in all models [31]. RMC-7977 treatment demonstrated sustained tumor inhibition for almost 90 days in mouse xenograft models. Direct targeting of activated RAS with RMC-7977 produced a distinct and superior antitumor activity profile compared to direct upstream and downstream suppression using SHP2 and MEK inhibitors in preclinical models of KRAS G12X mutant cancers [31]. The high level of antitumor activity of RMC-7977 may be attributed to the higher tumor levels compared with blood and to more effective inhibition of RAS oncogenic signaling in tumor cells than in normal cells [35], although the basis for this selectivity has not been well defined. Additionally, RMC-7977 showed slightly lower potency in WT cells and produced only partial WT RAS suppression compared with its stronger activity in KRAS G12X mutant cancer cells [31]. Nonetheless, this approach may address a critical limitation of mutant-specific KRAS inhibitors, whereby unchecked RAS isozymes may compensate and cause resistance.

Unlike the reactivation of the RAS pathway that commonly results from mutation-specific RAS inhibitors, broad inhibition of active RAS (RAS (ON)) directs tumors toward more limited mechanisms for developing resistance. Tumors may evade mutation-selective RAS suppression by reactivating RAS-MAPK signaling. This can occur through the emergence of second-site RAS mutations, the amplification of pathway components, or the activation of alternative signaling mechanisms. In a study conducted by Wasko et al., pancreatic tumors were found to relapse following initial exposure to RMC-7977. Sparse genome copy number variation analysis of RMC-7977-resistant tumors revealed focal copy number gains in the *MYC* gene, an oncogenic transcription factor that receives signals from the RAS and Wnt/β-catenin signaling pathways [35,36]. Another observation made by Wasko et al. was that the YAP–TAZ pathway has a role in supporting MYC-driven resistance to RMC-7977 [35]. Yes-associated protein (YAP)/transcriptional coactivator with a PDZ-binding domain (TAZ) are transcriptional activators; sustained activation of YAP/TAZ induces aberrant cellular proliferation by inducing other proto-oncogenic transcription factors, such as MYC [35]. Similar findings were observed in another study, demonstrating MYC amplification and enhanced activation of upstream RTK signaling through an HGF (hepatocyte growth factor)/MET (mesenchymal–epithelial transition) factor autocrine loop, both of which may contribute to RMC-7977 resistance [35,37]. Proteomics differential expression and gene set enrichment analysis revealed that the YAP-TAZ response was decreased by RMC-7977 in the sensitive line but not in the MYC amplified cell line, leading to the conclusion that MYC-driven resistance to RMC-7977 is facilitated by the YAP-TAZ pathway [35]. Consistent with these observations, the combination of RMC-7977 and the pan-TEAD (transcriptional enhanced associate domain) inhibitor, IAG933, showed additive or synergistic effects to inhibit MYC levels and other YAP targets [35]. Collectively, these findings suggest a possible strategy to overcome resistance to multi-selective RAS (ON) inhibition by dual RAS and MYC inhibition.

Another study conducted by Aust et al., in which a reporter-based screening approach was used to monitor KRAS downstream effector pathway activity, suggested that the emergence of resistance to broad-spectrum active-state RAS inhibition in CRC cell lines can arise from distinct mechanisms, resulting in varying degrees of reactivation of the MAPK- and PI3K-downstream pathways [38]. To further determine genetic mechanisms of KRAS inhibitor resistance, Long et al. performed KRASi-anchored CRISPR-Cas9 loss-of-function screens in KRAS G12D, KRAS G12C, KRAS G12R, and KRAS Q61H mutant PDAC cell lines using six KRASi to identify genes that modulate sensitivity to KRAS inhibition. Several hits from the screens, including EGFR, CK2, p110α and p110γ, and YAP were validated by combining targeted inhibitors with KRAS inhibitors [39]. They found that the EGFR inhibitor, erlotinib, synergized with RMC-7977 in KRAS Q61H mutant PDAC, which was otherwise less responsive to KRAS inhibitors by modifying ERK activity rebound. Based on analyses of a panel of RMC-7977-resistant PDAC cell lines and evaluation of inhibitor sensitivity and cross-resistance in the resistant lines, it was concluded that there will be many shared resistance mechanisms, as well as heterogeneous resistance mechanisms that vary by the mechanism of action of the inhibitor and KRAS mutational subtype [39]. Overall, the mechanisms of resistance to RMC compounds include both adaptive and acquired processes that enable tumor cells to bypass RAS pathway inhibition. Activation of the YAP/TAZ pathway contributes to both adaptive and acquired resistance [35], *c-myc* amplification represents a mechanism of acquired resistance [37], while adaptive resistance is also mediated by reactivation of MAPK and PI3K/AKT signaling pathways, often accompanied by epithelial-to-mesenchymal transition, along with activation of upstream receptor tyrosine kinase (RTK) signaling [35,37].

The interplay between RAS (ON) multi-selective inhibitors and the tumor microenvironment (TME) also influences resistance. It was demonstrated that RMC-7977 concentrations were higher in tumor tissues than in blood, indicating that its pharmacology can overcome the biophysical limitations imposed by a desmoplastic stroma. Based on this result, Wasko et al. predicted that daily or alternate-day dosing would yield an effective method to suppress RAS-MAPK signaling [35]. The impact of the TME on resistance to RMC-7977 was demonstrated through the induction of a desmoplastic response [40], significant reduction in tumor vascularity, and increase in CD4+ and CD8+ cells observed in the TME [37,40]. Moreover, the number of MHC class II-positive tumor cells increased, while the prevalence of monocytic and granulocytic myeloid-derived suppressor cells and M2 macrophages decreased [37].

There is also potential for RAS (ON) inhibitors to be effective for treating patients with brain metastases, as evidenced by RMC-6236’s ability to penetrate the blood–brain barrier and by its brain concentration increasing in a dose-dependent manner. Early-phase clinical trials are currently underway to assess the efficacy, tolerability, and optimal dosing of RMC-6236. RMC-6236 shows broad activity across multiple RAS mutants (beyond just G12C) and tumor types [41,42]. As described in an abstract presented at the European Lung Cancer Congress (ELCC) 2025, in NSCLC patients, RMC-6236 demonstrated durable clinical activity with a manageable safety and tolerability profile, as well as favorable dose intensity [43]. In a poster abstract presented at ASCO 2025, patients with previously treated RAS-mutant PDAC, RMC-6236 demonstrated a manageable safety profile and encouraging efficacy, as well as early and deep reductions in RAS-mutant ctDNA [44]. According to press releases from Revolution Medicines, the transition into a Phase III randomized trial in PDAC patients (ClinicalTrials.gov Identifier: NCT06625320; NCT07252232) represents the latest advancement toward possible regulatory approval, contingent upon favorable results [45,46]. However, the data currently available are from early-phase trials, which typically involve small or selected cohorts and employ single-arm designs. The safety profile described in the ELCC 2025 abstract, particularly concerning dermatologic toxicities, may restrict tolerable dosing in certain patients or tumor types [43]. Hence, the long-term durability of efficacy, potential for resistance, and safety of RMC-6236 in the clinic remain to be determined.

Positive early outcomes of pan-RAS inhibitors have raised the possibility of and expectations for the effectiveness of combination therapy. A study conducted by Araujo et al. emphasized the cumulative advantage of mutant-selective inhibitors, suggesting that, compared to monotherapy with a RAS (ON) multi-selective inhibitor, combination treatment could increase antitumor activity [40]. Evaluating RMC-6236 combination therapy, which is consistent with the idea that RAS (ON) pan-RAS inhibitors can be more effective when used in conjunction with other drugs, has been conducted in several studies. As described by a Revolution Medicines press release, a platform trial combining RMC-6236 with other agents in advanced gastrointestinal solid tumors harboring KRAS mutations is currently underway [47]. Preclinical studies suggest that RMC-7977, as a monotherapy or in combination with a RAS (ON) G12C mutant-selective inhibitor, demonstrates robust and durable antitumor activity against difficult-to-treat subsets of KRAS G12C-mutant NSCLC with primary or acquired KRAS G12C inhibitor resistance. Moreover, the trial identified a conserved mucinous transcriptional state that supports RAS inhibitor tolerance [40]. Additionally, Jiang et al. highlighted the synergy between RMC-6236 and immunotherapy, indicating promising avenues for combination therapy in NSCLC and other cancers [37]. This concept aligns with findings that tumor sensitization to immune-checkpoint blockade can be achieved by negating the immune-evasive effects of oncogenic *KRAS* [48,49].

Collectively, these data support the possibility that the efficacy of multi-selective tri-complex RAS (ON) pan-RAS inhibitors will exceed that of KRAS G12C inhibitors. In addition, their combination with other targeted drugs or immunotherapy may provide a significant advance for the treatment of patients who have developed resistance to mutant-specific KRAS inhibitors.

### 2.2. AN-9025

AN-9025 is another pan-RAS (ON) tri-complex inhibitor, which has been reported to exhibit high potency for inhibiting the proliferation of cancer cell lines harboring various RAS mutations [50]. Mechanistically, AN-9025 forms a RAS (ON)–CypA tri-complex like RMC-6236 but exhibits 3- to 8-fold stronger CypA binding affinity and a slower dissociation rate, suggesting a more stable binding complex. Consistent with this mechanism, AN-9025 exerted potent inhibition of proliferation across 26 RAS-mutant cancer cell lines from different tissue origins with sensitivity profiles comparable to RMC-6236 but approximately 100-fold greater in vitro potency. AN-9025 is orally bioavailable and elicited robust antitumor activity in multiple RAS-mutant xenograft models [50]. PK/PD studies highlight sustained inhibition of intratumoral DUSP6 levels, indicating prolonged target engagement and potential for intermittent dosing regimens [50]. Recruitment for the Phase 1 clinical trial of AN-9025 is currently in progress. Evidence is limited to abstracts; peer-reviewed binding/PK/PD data are pending [50].

### 2.3. GFH547

GFH547 is another pan-RAS (ON) tri-complex inhibitor that has been reported to recruit CypA and form a high-affinity complex that sterically disrupts RAS–effector interaction [51]. In preclinical models, GFH547 displayed high potency across 29 cancer cell lines representing a range of *KRAS*, *NRAS*, and *HRAS* mutations [51]. In addition, GFH547 suppressed the proliferation of cancer cells driven by *EGFR* or *FGFR2/3* amplification, mutation, or fusion, suggesting broader activity in the context of RTK activation [51]. Notably, GFH547 retained activity in models with adaptive or acquired resistance to switch II pocket (SIIP) KRAS inhibitors. In mouse tumor models, once-daily oral dosing of GFH547 at 1 mg/kg markedly suppressed ERK phosphorylation and induced tumor regression without overt toxicity [51]. GFH547 is currently in preclinical development. Evidence is limited to abstracts; peer-reviewed binding/PK/PD data are pending [51].

### 2.4. Cyclorasin B4-27

Cyclorasin B4-27 is a bicyclic peptide pan-RAS inhibitor optimized from a previously reported analog, cyclorasin B3, which itself was derived from the first-generation cyclorasin 9A5 by expanding the monocyclic scaffold into a more rigid bicyclic architecture [52,53,54]. These design refinements were reported to confer enhanced RAS-binding affinity, improved mutant RAS selectivity, and increased cell permeability [53,54]. Biochemically, cyclorasin B4-27 selectively binds to RAS-GTP with high affinity, showing a Kd value of 21nM for the KRAS G12V-GppNHp complex (non-hydrolysable GTP analog) [54]. Live-cell confocal microscopy confirmed that fluorescently labelled B4-27^FAM^ efficiently enters cells with a fraction of the compound localizing to the plasma membrane where RAS resides [54]. In KRAS-mutant transfected human embryonic kidney (HEK293T) cells, B4-27 demonstrates its capability to inhibit the binding of G12D and G12V to their downstream effector proteins, leading to suppression of the RAS signaling pathway and, ultimately, apoptosis in RAS-mutant cancer cells. B4-27 shows selective toxicity toward RAS-mutant cell lines, producing a single-digit µM EC_50_, while WT RAS cell lines display slightly higher EC_50_ values of 20 µM [54]. The authors suggest that the higher B4-27 endocytic uptake, lower RAS–effector binding affinity, and RAS addiction in mutant cells contribute to the observed selectivity for RAS-mutant cancer cells [54]. Despite being a peptide, B4-27 demonstrated metabolic stability, and its parenteral administration at low daily doses (1–5 mg/kg) effectively suppressed tumor growth in KRAS-mutant A549 and H358 mouse xenograft models over a 9-day treatment period, without apparent toxicity [54]. In the context of its developmental lineage, it is important to note that the first-generation cyclorasin 9A5 was later reported to exhibit false-positive RAS-binding behavior in orthogonal biophysical assays [55]. Although B4-27 is structurally distinct from 9A5 and was characterized using multiple biochemical binding and effector competition assays, independent validation using label-free biophysical methods remains to be reported [52,54,55].

### 2.5. YL-17231

YL-17231 is a reversible, small-molecule pan-RAS inhibitor that targets multiple oncogenic RAS mutants. Preclinical studies showed that YL-17231 suppressed P-ERK signaling in KRAS-mutant cancer cell lines and inhibited the proliferation of a broad panel of KRAS-, HRAS-, and NRAS-mutant cancer cell lines with an IC_50_ in the nanomolar range [56]. Beyond mutant targeting, YL-17231 also overcame resistance to the KRAS G12C inhibitor, sotorasib, and retained activity in cells harboring secondary KRAS mutations associated with acquired resistance. Mechanistically, YL-17231 induced G2 cell-cycle arrest and apoptosis. Daily oral administration of 4–8 mg/kg significantly inhibited tumor growth in the SW480 (KRAS G12V) CRC xenograft mouse model without causing significant body weight loss [56]. Evidence is limited to abstracts; peer-reviewed binding/PK/PD data are pending [56]. YL-17231 is currently being evaluated in a Phase I trial involving patients with advanced solid tumors harboring *KRAS*, *HRAS*, or *NRAS* mutations (ClinicalTrials.gov Identifier: NCT06096974) [57].

### 2.6. ADT-007 and Its Prodrug ADT-1004

A mechanistically distinct pan-RAS inhibitor, ADT-007, was recently described by Foote and colleagues as having high potency and selectivity for inhibiting the proliferation of a diverse panel of cancer cell lines harboring various *RAS* mutations or high levels of activated RAS resulting from mutations in upstream *RTKs* or *NF1*. Cancer cell lines with downstream *BRAF* mutations, as well as cells from normal tissues, were markedly less sensitive to ADT-007 treatment [58]. ADT-007 was reported to bind the nucleotide-free form of RAS (Apo RAS) that arises during GDP-GTP exchange. ADT-007 appears to block GTP binding to RAS, thereby disrupting RAS–effector interactions. The mechanism of binding appears to exploit a transitional conformation of RAS to block RAS activation while cycling from an inactive to an active state [6,58]. Treatment of KRAS-mutant CRC and PDAC cancer cell lines with ADT-007 resulted in a marked reduction in activated RAS-GTP levels, followed by decreased phosphorylation of RAF, MEK, ERK, and AKT.

The reduction in MAPK and AKT signaling by ADT-007 was closely associated with G2/M cell-cycle arrest (mitotic arrest), as demonstrated by the accumulation of phospho-histone H3, followed by apoptosis induction [58]. While the pan-KRAS inhibitor, BI-2865, and the pan-RAS inhibitor, RMC-6236, effectively inhibited the proliferation of KRAS-mutant pancreatic cells, neither induced apoptosis, whereas ADT-007 did. This difference was readily apparent in colony formation assays, in which BI-2865 and RMC-6236 reduced the colony number only partially, while ADT-007 caused near-complete inhibition, despite testing all three compounds at their IC_50_ concentrations. The pan-RAS inhibitory activity of ADT-007 was also reported to confer activity against tumors with mixed RAS mutations (e.g., *KRAS* and *NRAS*), highlighting a distinct advantage over pan-KRAS inhibitors, as discussed below [58,59,60,61]. Consequently, ADT-007 may be less susceptible to intratumoral heterogeneity and secondary mechanisms that cause resistance to mutant- or isoform-specific KRAS inhibitors.

The basis for ADT-007’s selectivity in differentially blocking the growth of RAS-mutant cancer cells vs. RAS WT cancer cells or normal cells appears to involve a unique mechanism of action. ADT-007 contains a phenolic hydroxyl group, which was reported to be essential for RAS binding, given that analogs with a methoxy substitution lack such selectivity. The phenolic hydroxy moiety also serves as a substrate for glucuronide conjugation by UDP-glucuronosyltransferases (UGTs), which would sterically interfere with ADT-007 binding to RAS. UGTs are well known to be expressed in the liver, but also non-hepatic cells, although Foote et al. reported that UGT isozymes have low expression in cancer cells harboring KRAS mutations. Consequently, ADT-007 binds RAS in RAS-mutant cancer cells expressing low levels of UGTs to potently inhibit growth, whereas ADT-007 would not impact cells from normal tissues expressing high levels of UGTs [58].

To overcome first-pass metabolism, an orally bioavailable prodrug of ADT-007, ADT-1004, was developed. Masking the glucuronidation site with a carbamate moiety, ADT-1004 allows for stable systemic delivery and release of ADT-007, the active compound [62].

In murine models of RAS-driven PDAC, ADT-1004 demonstrated robust and durable antitumor activity at well-tolerated doses. Oral dosing maintained therapeutic plasma levels of ADT-007 and produced significant tumor growth inhibition, which correlated with reduced activated RAS levels and ERK signaling [62]. ADT-1004 was effective in PDX models of pancreatic cancer harboring KRAS mutations (G12D, G12V, G12C, and G13Q) but did not affect the growth of tumors established from the WT RAS BxPC3 pancreatic tumor cell line, which is driven by a downstream *BRAF* mutation. These results highlight both the broad activity of ADT-1004 and selectivity for RAS-driven cancers. Treated tumors exhibited increased infiltration of CD4+ and CD8+ T cells, as well as activation of M1 macrophages and dendritic cells, indicating that ADT-1004 enhances antitumor immunity. Notably, ADT-1004 also demonstrated superior efficacy compared with sotorasib and adagrasib in pancreatic cancer cell lines that developed resistance to these mutant-specific KRAS inhibitors. Overall, these findings support the clinical development of ADT-1004, given its strong antitumor activity and potential for a wide therapeutic window. Treatment strategies could combine ADT-1004 with immune checkpoint inhibitors to enhance or broaden the efficacy of immunotherapy based on its capacity to increase immune cell infiltration to transition the tumor microenvironment from “cold” to “hot” [58,62].

### 2.7. ADT-030

ADT-030 is a second-generation analog of ADT-007 that suppresses both RAS-mediated MAPK/AKT signaling and Wnt/β-catenin transcriptional activity, with the latter resulting from inhibiting phosphodiesterase 10A [63]. Notably, ADT-030 also reduced Wnt/β-catenin-driven transcription of *MYC* [64]. Hence, ADT-030 may overcome resistance to other RAS inhibitors driven by *MYC* upregulation [35]. ADT-030 simultaneously induces immunogenic cell death in cancer cells, promoting dendritic cell antigen uptake and maturation [65]. Thus, ADT-030 has a significant effect on both cancer cells and on the tumor immune microenvironment, with the potential for monotherapy or use in combination with immunotherapies for the treatment of mutant RAS cancers.

## 3. Pan-KRAS Inhibitors

As efforts to develop pan-RAS inhibition continue to advance, substantial progress has also been made in the development of isoform-specific pan-KRAS inhibitors. These inhibitors target multiple *KRAS* mutants, as well as wild-type *KRAS*. These molecules are predicted to have a more manageable side effect profile by sparing normal HRAS and NRAS signaling. Preclinical studies have shown that normal cells do not exhibit dependence on specific individual RAS isoforms, especially KRAS, and that compensatory signaling by HRAS or NRAS isoforms may be sufficient to maintain homeostasis. Hence, KRAS isoform-selective targeting may provide a more advantageous toxicity profile than pan-RAS inhibitors [30]. Clinical evidence regarding the tolerability of KRAS-specific targeting in patients is still pending. However, specific KRAS targeting may allow for the possibility of resistance developing through compensatory signaling via wild-type NRAS or HRAS isoforms. As a class, pan-KRAS inhibitors have demonstrated broad activity across various cancers with KRAS mutations, and many have advanced into clinical trials, although it is unclear whether pan-KRAS inhibitors will be able to escape resistance as expected for pan-RAS inhibitors.

### 3.1. BI-2865, BI-2493, and BI 3706674

BI-2865 is a pan-KRAS inhibitor that targets multiple KRAS mutants, including G12C, G12D, G12V, and G13D, by reversibly binding RAS in its GDP-bound inactive state [66]. Biochemical assays demonstrate potent inhibition of RAS by BI-2865, with IC_50_ values in the low nanomolar range and high selectivity for inhibiting the proliferation of cancer cells with mutant KRAS, but not with HRAS or NRAS mutations. In KRAS-mutant cancer cell lines, BI-2865 significantly suppressed downstream MAPK signaling as evidenced by a reduction in ERK phosphorylation, while showing limited effects on MAPK signaling in WT KRAS cancer cell lines [66]. BI-2493, a structural analog of BI-2865, was optimized for potency, metabolic stability, and oral bioavailability by introducing a spirocycle into the chemical scaffold [67]. Mouse studies have shown that BI-2493 can effectively inhibit tumor growth in various KRAS-mutant and WT KRAS-amplified mouse xenograft models without causing significant body weight loss [67,68]. Treatment caused strong suppression of both ERK phosphorylation and *DUSP6* mRNA expression [68]. BI 3706674, a compound with improved potency and optimized drug metabolism and PK properties compared to BI-2865 and BI-2493, was later developed and has entered a Phase I clinical trial to evaluate PK/PD and safety in patients with gastroesophageal cancers carrying WT KRAS amplifications (ClinicalTrials.gov Identifier: NCT06056024) [69,70].

### 3.2. BBO-11818

BBO-11818 is an orally bioavailable pan-KRAS inhibitor that selectively and non-covalently binds to both the active and inactive conformations of mutant KRAS [71]. This pan-KRAS inhibitor blocks RAS-RAF signaling by stabilizing the unproductive “dark” state 1 conformation of KRAS, thus disrupting the association of GTP-bound KRAS with RAF1 [71,72]. Cell-based assays demonstrated low nanomolar potency of BBO-11818 in suppressing MAPK signaling and the proliferation of KRAS-mutant cells [71]. BBO-11818 exhibited favorable PK/PD and antitumor activity in mouse tumor xenograft models in KRAS-mutant NSCLC, PDAC, and CRC CDX models [71]. These findings have been communicated primarily through conference presentations [71], and peer-reviewed data describing the compound’s efficacy, PK, or binding details have not yet been published.

### 3.3. LY4066434

LY4066434 is an orally administered small-molecule pan-KRAS inhibitor, which exhibits high selectivity over NRAS and HRAS [73]. The compound has demonstrated robust antitumor activity in KRAS-mutant PDX mouse models of NSCLC, CRC, PDAC, and gastric cancers [73]. Additionally, LY4066434 inhibited the growth of KRAS-mutant metastatic brain tumors in orthotopic mouse models of NSCLC [73]. The combination of LY4066434 with chemotherapies achieved greater antitumor activity than single-agent treatment in various KRAS-mutant cancer models [73]. A Phase I clinical trial involving patients with KRAS-mutant tumors is currently open for enrolment (ClinicalTrials.gov Identifier: NCT06607185) [74].

### 3.4. AMG 410

AMG 410 is a reversible pan-KRAS inhibitor that binds to the same allosteric pocket targeted by sotorasib, a KRAS G12C inhibitor [75]. AMG 410 demonstrated 100-fold greater selectivity toward KRAS over HRAS and NRAS and exhibited potent antiproliferative activity in KRAS-mutant cell lines [75]. Notably, AMG 410 acts as a dual inhibitor, targeting both GTP (on)- and GDP (off)-state (K_D_ (GDP-state) = 1 nM; K_D_ (GTP-state) = 22 nM)), enabling sustained blockade of KRAS signaling and inhibition of proliferation in WT KRAS-amplified cancer cell lines [75]. AMG 410 demonstrated potent antitumor activity, which was associated with reduced phosphorylated ERK levels, across CRC, PDAC, and NSCLC CDX and PDX mouse models harboring diverse KRAS mutations, including G12C, G12D, G12V, and G13D. AMG 410 also shows good tolerability and enhanced efficacy in combination with targeted therapies or immunotherapy, supporting the potential of an HRAS- and NRAS-sparing pan-KRAS inhibitor in combination treatment settings. The compound has now advanced into Phase I clinical testing (ClinicalTrials.gov Identifier: NCT07094113) [76].

### 3.5. JAB-23E73

JAB-23E73 is another pan-KRAS inhibitor that targets both the GTP- and GDP-bound forms of KRAS [77]. Studies have shown that JAB-23E73 suppresses ERK phosphorylation and inhibits the proliferation of cancer cell lines harboring mutant KRAS or amplified WT KRAS with nanomolar IC_50_ values. A PK/PD study of JAB-23E73 demonstrated a correlation between tissue and plasma levels of the drug and inhibition of ERK phosphorylation in tumors from treated mice [77]. Oral treatment exhibited potent antitumor activity and tolerability across multiple mouse tumor models harboring various *KRAS* mutations, including KRAS G12D, G12V, G12A, or G13D mutations [77]. While these results have not been peer-reviewed, JAB-23E73 is currently in Phase I clinical trials for patients with advanced solid tumors and KRAS alterations (ClinicalTrials.gov Identifier: NCT06959615 (China) and NCT06973564 (U.S.)) [78,79].

### 3.6. ERAS-4001

ERAS-4001 is a novel pan-KRAS inhibitor that binds both WT KRAS and multiple KRAS G12X mutants, blocking downstream interaction with RAF proteins in both the GTP- and GDP-loaded states with single-digit nanomolar potency [80]. Biochemical studies show strong selectivity for KRAS over HRAS and NRAS. In cellular assays, ERAS-4001 inhibited ERK phosphorylation and suppressed growth across WT KRAS-amplified and KRAS G12X mutant NSCLC, PDAC, and CRC cell lines with nanomolar IC_50_ values [80]. Oral administration of ERAS-4001 in mice achieved dose-dependent plasma exposure, reduced P-ERK levels, and produced antitumor activity, including 77% tumor growth inhibition in KRAS G12D xenograft models and tumor regression in G12V xenograft models [80]. Though these results were not peer-reviewed, ERAS-4001 is currently the subject of a Phase I clinical trial to assess its safety in patients with advanced or metastatic solid tumors (ClinicalTrials.gov Identifier: NCT07021898) [81].

## 4. Human-Relevant New Methods for Evaluating Pan-RAS Inhibitors

The clinical promise of pan-RAS inhibitors depends not only on their pharmacological properties but also on the fidelity of the preclinical platforms used to evaluate them. Most potency and selectivity data for this drug class have been generated in two-dimensional (2D) monolayer cultures. However, these platforms lack the three-dimensional (3D) architecture, diffusion gradients, and tumor microenvironment complexity of human solid tumors [82,83,84]. For a drug class in which the therapeutic index between mutant RAS-dependent cancer cells and normal tissues is the central translational question, these limitations are consequential.

Three-dimensional bioprinted tumor models represent an emerging class of New Approach Methodologies (NAMs) that address these gaps. Bioprinted ex vivo slice tissue (BEST) constructs, fabricated using precision additive manufacturing, recapitulate 3D cellular organization and tunable extracellular matrix composition while enabling high-throughput evaluation through orthogonal readouts, including multiplane high-content imaging and metabolic activity assays [85,86]. The utility of such platforms for pan-RAS inhibitor evaluation was recently demonstrated by De Nobrega et al., who assessed ADT-007 in KRAS-mutant (HCT-116 human CRC) and WT RAS (HT29 human CRC) BEST [86]. Notably, disruption of 3D tumor cell organization was observed at concentrations below 2D-derived IC_50_ values, indicating that architecturally relevant phenotypic readouts can detect drug activity that is not readily apparent in conventional monolayer assays and even in murine models.

Patient-derived BEST incorporating autologous tumor and stromal populations could further enable evaluation of pan-RAS inhibitors within a human-relevant context, supporting assessment of stromal remodeling, resistance evolution, and mechanism-informed combination screening. More broadly, NAMs such as 3D bioprinted constructs, patient-derived organoids, and microfluidic tumor-on-chip systems are increasingly compatible with high-throughput screening workflows, including standard multiwell plate formats and automated liquid handling, lowering barriers to adoption in drug discovery pipelines. Each platform, however, presents distinct trade-offs. Organoids preserve tumor heterogeneity and are amenable to patient-derived biobanking but generally fall short in recapitulating the tissue architecture and stromal and immune cell compartments. Microfluidic tumor-on-chip systems can incorporate vascularization and fluid flow, but this added complexity increases fabrication demands and run-to-run variability, reduces throughput, and may introduce confounding variables that complicate the interpretation of drug-specific effects. Bioprinted constructs such as BEST offer tunable matrix composition and scalability for high-throughput screening but currently lack vascularization. No single platform fully recapitulates the integrated tumor microenvironment, underscoring the need for complementary deployment across the preclinical pipeline. As pan-RAS inhibitors progress toward clinical translation, integrating high-fidelity 3D tissue-engineered models into preclinical workflow will be essential for maximizing therapeutic impact and reducing late-stage attrition.

## 5. Summary

**Figure 2:**
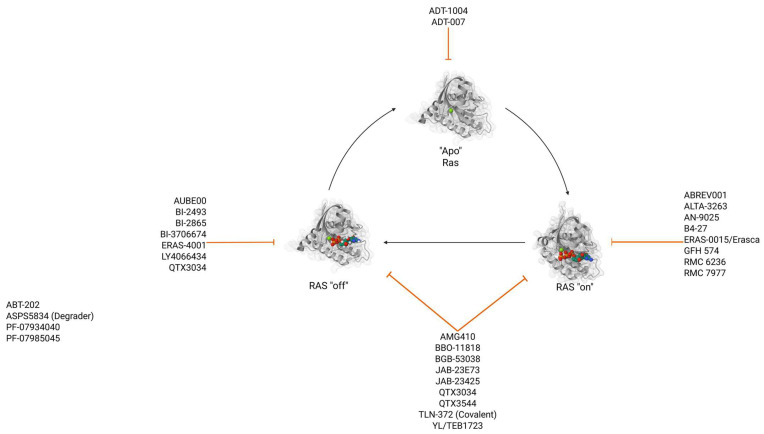
Depicted is a crystal ribbon structure of RAS with a transparent volume (gray) bound to either GTP (RAS “on”; GTP = colored ligand), GDP (RAS “off”; GDP colored ligand), or nucleotide-free RAS (“Apo” RAS; magnesium ion = green dot). The mechanism of action of the pan-KRAS or pan-RAS inhibitors is depicted by what state of RAS they act on (blunted arrows = inhibition).

This review of pan-RAS and pan-KRAS inhibitors in preclinical or clinical development is based primarily on peer-reviewed publications and non-peer-reviewed conference abstracts submitted by the pharmaceutical companies developing the inhibitors. These inhibitors, along with additional emerging inhibitors for which limited public information is available, are listed in Table 1 and Figure 2. As only KRAS G12C-mutant-specific inhibitors are FDA-approved, their use is limited to patients diagnosed with KRAS G12C-mutant lung and metastatic CRC. In addition, their efficacy is limited by multiple mechanisms of resistance, some of which have been identified, while other mechanisms remain unknown. Although preclinical evidence suggests that pan-RAS or pan-KRAS inhibitors may evade these mechanisms of resistance, additional research is needed to determine which inhibitors and mechanisms of action will have superior efficacy as monotherapies and/or in combination with standard treatments.

## 6. Conclusions

Targeting of one of the historically most “undruggable” oncoproteins, RAS, has reached a major milestone: the FDA approval of mutant-specific KRAS inhibitors and the rapid emergence of pan-RAS and pan-KRAS inhibitors. By concurrently inhibiting all RAS isoforms and mutant variants, pan-RAS and pan-KRAS inhibitors have potential for broader therapeutic use and the capacity to overcome resistance caused by RAS mutation heterogeneity, compensatory signaling from WT RAS isoforms, and possibly other mechanisms that have yet to be discovered. Collectively, the preclinical and early clinical evidence reviewed here demonstrates that broad RAS inhibition is achievable with better-than-expected tolerability and activity across diverse RAS mutations and tumor types.

The challenges moving forward for pan-RAS and pan-KRAS inhibitors include achieving adequate selectivity to inhibit tumor growth while minimizing toxicities to normal tissues and optimizing combination therapy with standard-of-care chemotherapy or immunotherapy, including CAR-T cell therapy. Overall, pan-RAS and pan-KRAS inhibitors represent a significant breakthrough in oncology. Their rapid progression into clinical trials, driven by sophisticated medicinal chemistry approaches and screening tools, is rapidly advancing, though additional basic research is needed to determine which mechanisms of action offer the greatest potential to evade resistance, ultimately guiding the possibilities for clinical use of these promising agents.

## Figures and Tables

**Table 1:** Clinical trial data were retrieved from ClinicalTrials.gov.

Mechanism	Inhibitors	Development Stage
pan-RAS	ADT-1004	Preclinical [62]
	AN-9025	Phase I; NCT07252479 [50]
	Cyclorasin (B4-27)	Preclinical [54]
	ERAS-0015/Erasca	Phase I; NCT06983743 [87]
	GFH547	Preclinical [51]
	RMC-6236	Phase 3; NCT06625320 *
	YL/TEB-17231	Phase I; NCT06096974 [57]
pan-KRAS	ABREV001	Preclinical
	ABT202	Preclinical
	ALTA3263	Phase I; NCT06835569 [88]
	AMG410	Phase I; NCT07094113 [76]
	ASP5834	Phase I; NCT07094204 [89]
	AUBE00	Phase I; NCT07030959 [90]
	BBO-11818	Phase I; NCT06917079 [91]
	BGB-53038	Phase I; NCT06585488 [92]
	BI 3706674	Phase I; NCT06056024 [70]
	ERAS-4001	Phase I; NCT07021898 [81]
	JAB-23E73	Phase I; NCT06959615 [78], NCT06973564 [79]
	LY4066434	Phase I; NCT06607185 [74]
	PF-07934040	Phase I; NCT06447662 [93]
	PF-07985045	Phase I; NCT06704724 [94]
	QTX3034	Phase I; NCT06227377 [95]
	TLN-372	Phase I; NCT07204340 [96]

* Ten additional Phase I, II or III clinical trials are also in progress.

## Data Availability

No new data were created or analyzed in this study. Data sharing is not applicable to this article.
